# Family caregiver’s willingness to care from the perspective of altruism

**DOI:** 10.3389/fpubh.2023.1237241

**Published:** 2023-11-23

**Authors:** Jun Li, Jing-Chun Zhan, Cui-Hua Xie, Shao-Yong Han

**Affiliations:** ^1^School of Social Development, East China University of Political Science and Law, Shanghai, China; ^2^School of Computing, Universiti Teknologi Malaysia, Johor Bahru, Malaysia; ^3^School of Economics and Management, Wenzhou University of Technology, Wenzhou, China; ^4^Postdoctoral Scientific Research Workstation, Bank of Zhengzhou, Zhengzhou, China

**Keywords:** altruism, willingness to care, family caregivers, older adults with functional disabilities, caregiver burden

## Abstract

**Objectives:**

The willingness of family members to take care of older relatives directly affects the quality of life of disabled older adults, so it is necessary to understand the status quo of willingness to care and its influencing factors. This has been extensively studied in other countries, but, it is rarely studied in China. Based on the theory of altruism, employing a unique sample from Shanghai, China in 2017 and 2022, we attempt to reveal the influencing factors of the care willingness of family caregivers during the transition period.

**Methods:**

To measure caregiver burden and functional disability of the care recipient, we employ the Zarit Burden Interview (ZBI) and the Barthel Index, respectively. Then we utilized the ordinary least squares (OLS) methodology and estimated four regression models. Models 1, 2, and 3 examined the impact of the variables of the caregiver burden, responsibility and love, and the quality of the caregiver-caregiver recipient relationship, respectively, on family caregivers’ willingness to care. Model 4 was the full model. To testify whether the caregiver burden is likely to act as a mediator, path analysis was used, and the path was adjusted and verified.

**Results:**

According to the survey, in Shanghai, only half of the caregivers had a very high care willingness to care for disabled older relatives, while nearly one-tenth of the caregivers had a low willingness. It was the caregiver burden rather than the functional disability of older adults that harms family caregivers’ willingness to care. Responsibility and caring out of love were positively related to care willingness. Relationship quality was the most important influencing factor, explaining 10.2% of the variance in care willingness. Path analysis demonstrated that responsibility, caring out of love, and relationship quality directly and through the mediation of caregiver burden indirectly affected care willingness.

**Conclusion:**

Our results revealed that reciprocal altruism presented by the quality of the caregiver-care recipient relationship had a significantly positive impact on family caregivers’ willingness to care. In addition, the caregiver burden was found not only directly affected care willingness, but also acted as a mediator. To promote the perfection of laws and policies, comprehensive samples of different types of cities should be included and the measurement of key variables could be further improved in future studies.

## 1 Introduction

The population over the age of 65 in China reached 209.78 million—14.9% of the total population in 2022 (1). It is predicted that there will be 97.5 million disabled older people in China by 2050 (2). Caring for older adults with functional disabilities has become an arduous task (3). Willingness to care is a critical factor that could influence caregiving decisions. Generally speaking, the heavier the caregiver burden, the weaker the willingness to care. Lévesque et al. (4) pointed out that institutional care usually occurs when the health of older relatives deteriorates significantly, and caregivers are exhausted. Gaugler et al. (5) also found that the stress and burden shouldered by caregivers not only affect their health but also result in premature institutionalization of their older relatives. Most studies focus more on the impact of objective burden (i.e., older people’s ability to perform ADL) on family caregivers’ willingness to care (6). However, the caregiver burden is influenced by many factors and needs to be assessed by the caregiver himself or herself, that is, subjective burden (7). Some studies have shown that the subjective burden affects the continuity of family care more than the objective burden (8). Consequently, the caregiver burden referred to below mainly refers to the subjective burden, and Hypothesis 1 is proposed: the heavier the caregiver burden, the lower the willingness to care.

From the perspective of psychology, willingness to care is closely related with altruism (9). Altruism includes genetic altruism and reciprocal altruism. Genetic altruism refers to engaging in altruistic behavior that benefits close relatives (10). The US evolutionary biologist Robert Trivers proposed reciprocal altruism in 1971 (11), altruistic behavior undertaken with an expectation of reciprocation. Many population biologists insist that, except for altruism to close relatives, some seemingly altruistic human actions are actually aimed at reciprocity (12). Caring for older people inevitably entails stress, known as the caregiver burden. The return of caregivers to the old people’s past efforts, and the good relationship between caregivers and the old people can meet the psychological needs of caregivers, so as to realize a kind of reciprocity.

From an altruistic point of view, responsibility, love and relationship quality are conducive to strengthening willingness to care. Light et al. (13) found that family members most often expressed a strong desire and sense of responsibility to care for older adults. Many studies proved that the higher the family members’ filial piety identity is, the higher willingness they have to care for their older adults (14–16). Li et al. (17) found that the decline of filial piety would lead to a reduction in family members’ willingness to care. Chien and Wu (6) found that the more filial piety adult children identify with, the more willing they are to care for their disabled parents. Therefore, Hypothesis 2 can be raised: the higher the family caregiver’s sense of responsibility for the care recipient, the higher the willingness to care.

Love can also explain family caregivers’ willingness to care to some extent. Bull and McShane (18) argued that most family members provided care to older adults primarily out of love and dedication. Baider and Surbone (19) also found that those who cared for older people at home usually did so out of genuine love. Similar findings have been found in many studies (20, 21). So, we put forward Hypothesis 3: family caregivers motivated by love are more willing to provide care than those not motivated by love.

The relationship between the caregiver and the care recipient includes spousal relationship and parent–child relationship. Mercier et al. (22) studied 87 mother-daughter and 70 father-daughter pairs, and found that the better the quality of relationship, the more the daughters felt obligated to care for their older parents. A Chinese study also found good intergenerational relationships can provide a vital source of motivation and spiritual support for children to look after their older parents (23). As for older adults with functional disabilities, Chien and Wu (6) found that the willingness of adult children to care for their disabled parents is significantly influenced by intergenerational relationships. Therefore, we can put forward Hypothesis 4: the better the quality of the relationship between the caregiver and the care recipient, the higher the willingness to care.

Lawrence et al. (24) found that good relationships between family caregivers and their older relatives were thought to be effective in coping with pressure and counteracting the negative consequences of care. Based on Hypothesis 1, reducing the caregiver burden will enhance care willingness to some extent. Therefore, the impact of relationship quality on care willingness may be either direct or indirect through the mediation of caregiver burden. Similarly, if the caregiver has a strong sense of responsibility for the care recipient or caring for him/her out of love, the caregiver’s burden may be reduced, thus leading to an increased willingness to care. Therefore, Hypothesis 5 is proposed: caregiver burden is a mediator between independent variables (responsibility for the care recipient, caring out of love, the quality of the caregiver-care recipient relationship) and dependent variable (family caregivers’ willingness to care).

Due to the lack of data, there have been few empirical studies on Chinese family caregivers’ willingness to care. Therefore, it is necessary to conduct empirical research (explanatory studies specifically) to understand more about it. Employing a unique sample from Shanghai, China, this current study attempts to reveal the influencing factors of the care willingness of family caregivers during the transition period. Based on empirical findings, family care support policies can be further improved to make it sustainable.

## 2 Materials and methods

### 2.1 Ethical consideration

We focus on caregiving within a family context. The care recipients are all disabled older adults, and the caregivers are mainly their family members, including spouses and adult children. In 2017 and 2022, the study involving human participants was supported by the Shanghai Municipal Health Commission and was reviewed and approved by the Institutional Review Board of East China University of Political Science and Law (Protocol #16BSH137).

### 2.2 Participants

We conducted a sample survey of family caregivers and their disabled older relatives in Shanghai. After informed consent, paper questionnaires were completed by family caregivers and their disabled older relatives, so, two questionnaires were collected from one family. We obtained complete information on family care, including information about family caregivers and their disabled older relatives, their interactions, as well as general family situations, which helped us to identify factors influencing family caregivers’ willingness to care. Due to the large scope of Shanghai, we chose our participants by implementing three-stage (district, town, and family) sampling. The total number of disabled older adults from 10 districts (the first stage sampling), 18 towns (the second stage sampling) was no more than 10,000. We completed a survey of 30 households (the third stage sampling) in each town due to the hospitalization of some disabled older adults and the refusal of some family caregivers to the survey. The distribution of samples in central urban areas (325 pairs) and suburbs (210 pairs) is basically the same as the distribution of population. Among the investigated caregivers, there are 188 spouse caregivers and 347 child caregivers.

### 2.3 Measures

In this paper, willingness to care is the dependent variable, which is assumed to be influenced by five independent variables – functional disability of the care recipient, caregiver burden, responsibility, caring out of love, and quality of the caregiver–care recipient relationship.

#### 2.3.1 Willingness to care

In the questionnaire, we asked family caregivers, “To what extent are you willing to look after the older adult at present?” The total score of willingness to care ranges from 0 to 10. If family caregivers had high care willingness, the score was higher, and vice versa.

#### 2.3.2 Functional disability of the care recipient

The Barthel Index was adopted to evaluate older people’s ability to perform ADL (25). The index comprises 10 activities, such as feeding, dressing, and bathing. The maximum value of the index is 100, and the minimum value is 0. The smaller the score is, the higher the level of functional disability of older people. The Cronbach’s alpha of the Barthel Index is 0.903.

#### 2.3.3 Caregiver burden

The Zarit Burden Interview (ZBI) was used to assess caregiver burden. The ZBI is made up of four dimensions, including health status, mental state, economic status, and social life, with a total of 22 items. Each item is rated from 0 to 4 (0 = never; 4 = always). The value of the scale ranges from 0 to 88. The larger the score is, the heavier the caregiver burden (26). The Cronbach’s alpha of ZBI is 0.878.

#### 2.3.4 Responsibility

The responsibility of the primary caregiver for the care recipient is measured through a question, that is, “Do you think you have the responsibility to care for the older adult?” There are five options, from “strongly disagree” (=1) to “strongly agree” (=5).

#### 2.3.5 Caring out of love

In the questionnaire, “what’s the primary reason you care for the older adult?” There are eight options in total, and only one option is related to affection for older relatives: “I have affection for him and it makes me happy to take care of him.” Thus, these eight options can be divided into two categories, namely “caring out of love” (=1) and “caring not out of love” (= 0).

#### 2.3.6 Quality of the caregiver–care recipient relationship

To measure the quality of the relationship between primary caregivers and older adults, it is necessary to have information not only from the caregiver’s perspective, but also from the older adult’s perspective. In the questionnaire, caregivers and older people were asked to assess their relationship, respectively (1 = very bad; 5 = very good).

#### 2.3.7 Demographic characteristics

The following demographic variables were used as control variables in this study, including caregiver gender (1 = male, 0 = female), age (in years), completed formal education (in years), physical health (1 = very bad; 5 = very good), and the relationship between caregivers and older adults (1 = spousal relationship; 0 = parent–child relationship).

### 2.4 Statistical analysis

Four regression models were estimated by using ordinary least squares (OLS) method. Model 1 investigated the influence of caregiver burden on family caregivers’ willingness to care. Model 2 demonstrated the impact of responsibility and love, and Model 3 showed the effect of the quality of the caregiver–care recipient relationship. Model 4 was the full model, showing the effect of all independent variables on family caregivers’ willingness to care. The same control variables were included in all models.

## 3 Results

Descriptive statistics were performed for all variables, as shown in Table 1. The average score of care willingness was 8.55. Out of the full score of 10, 54.91% of family caregivers gave a score of 9 or above, and 35.65% gave a score between 7 and 8, 6 and below accounted for 9.44%. The data suggest that a certain proportion of family caregivers are not willing to take care of their older relatives.

**Table 1:** Descriptive statistics of study participants (*N* = 532).

Variables	*M* (*SD*)	Ranges
Willingness to care	8.55/1.59	0–10
ADL (CR)	51.18/40.60	0–100
Caregiver burden	29.10/17.38	0–88
Responsibility	4.32/1.08	1–5
Caring out of love	0.08/0.27	0–1
CG’s relationship to CR	4.11/0.90	1–5
CR’s relationship to CG	4.18/0.72	1–5
Spouse caregivers	0.35/0.48	0–1
Male (CG)	0.42/0.49	0–1
Age (CG)	65.13/12.39	25–97
Education (CG)	11.25/3.58	0–19
Physical health (CG)	3.08/0.86	1–5

CG denotes variables pertaining to caregivers. CR indicates variables pertaining to care recipients. ADL, activities of daily living.

### 3.1 Influencing factors of willingness to care

The statistical results about factors influencing family caregivers’ willingness to care are demonstrated in Table 2.

**Table 2:** OLS regression model of predictors of care willingness (*N* = 532).

Variables	Model 1	Model 2	Model 3	Model 4
ADL (CR)	−0.003			−0.002
	(0.002)			(0.002)
Caregiver burden	−0.023**			−0.014**
(0.005)			(0.005)
Responsibility		0.224**		0.133
		(0.072)		(0.069)
Caring out of love		0.679**		0.429
		(0.228)		(0.222)
CG’s relationship to CR			0.374**	0.287**
			(0.092)	(0.089)
CR’s relationship to CG			0.288*	0.215
			(0.123)	(0.122)
Spouse caregivers	0.069	−0.006	0.119	0.095
	(0.210)	(0.217)	(0.214)	(0.212)
	Male (CG)	−0.109	0.019	−0.032	−0.034
		(0.133)	(0.138)	(0.130)	(0.130)
	Age (CG)	0.006	0.007	0.005	0.003
		(0.010)	(0.010)	(0.010)	(0.010)
	Education (CG)	−0.002	−0.012	−0.023	−0.007
	Physical health (CG)	(0.022)	(0.022)	(0.022)	(0.021)
		0.026	0.157	0.114	−0.014
		(0.087)	(0.087)	(0.082)	(0.085)
		Constant	8.936***	6.689***	5.335***	6.160***
		(0.778)	(0.786)	(0.824)	(0.972)
R^2^		0.087	0.053	0.102	0.154

Robust standard errors in parentheses. CG denotes variables pertaining to caregivers. CR denotes variables pertaining to care recipients. OLS, ordinary least squares; ADL, activities of daily living. *p < 0.05; **p < 0.01; ***p < 0.001.

Model 1 presented the correlations between caregiver burden and care willingness. The disabled older people’s ability to perform ADL had no significant impact on care willingness, which implied that family caregivers’ willingness to care was not related to the functional disability of the care recipient. However, caregiver burden was negatively associated with care willingness (*p* < 0.01), indicating that the heavier the caregiver burden, the lower the care willingness. Hypothesis 1 was verified.

The influence of responsibility and love on care willingness was demonstrated in Model 2. Care willingness was higher for those caregivers who had a strong sense of responsibility (*p* < 0.01) and for those caregivers who took care of older relatives out of love (*p* < 0.01). Hypothesis 2 and 3 were verified.

Model 3 showed the effect of relationship quality on care willingness. According to the evaluation of relationship quality by caregivers and care recipients, the statistical results showed that there was a positive correlation between relationship quality and care willingness (*p* < 0.01 and *p* < 0.05, respectively), indicating that a better caregiver-care recipient relationship was related to a higher willingness to care. Hypothesis 4 was verified.

It was demonstrated in Model 4 that 15.4% of the variance in care willingness was explained. Although three independent variables were no longer statistically significant, the caregiver burden and the caregiver’s relationship to the care recipient still had a significant effect on family caregivers’ willingness to care.

### 3.2 Mediating effect of caregiver burden

**Figure 1:**
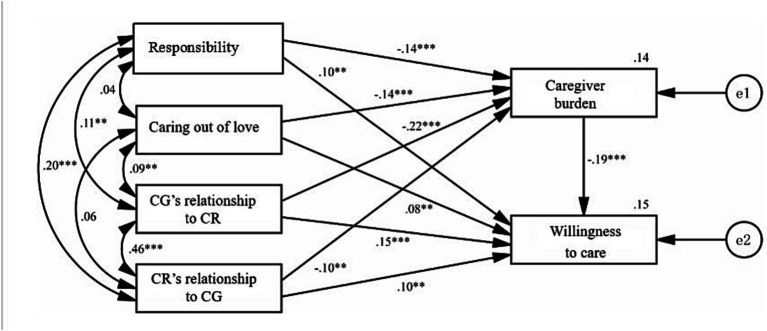
Path analysis coefficients of the model.

It was found that the responsibility and love of family caregivers for disabled older relatives, and the quality of the caregiver-care recipient relationship have significant effects on the caregiver burden (24, 27, 28) in previous studies. We found that the caregiver burden has significant effects on care willingness, so we guessed that the caregiver burden is likely to act as a mediator. Therefore, we used path analysis, adjusted and verified the path, and finally got a statistically significant model, as shown in Figure 1. The Compared Fit Index (CFI > 0.9, indicating that the model has an excellent fitting degree) is 1.000. The Root Mean Square Error of Approximation (RMSEA <0.1, showing that the model has an excellent fitting degree) is 0.000. Consequently, it can be concluded that the model has an excellent fitting degree. The direct effect, indirect effect, and total effect of care willingness with caregiver burden as the mediator are shown in Table 3.

**Table 3:** Direct, indirect, and total effect of care willingness with caregiver burden as the mediator.

Independent variables	Direct effect	Indirect effect	Total effect
Responsibility	0.149*	0.040**	0.189**
Caring out of love	0.480*	0.156**	0.636**
CG’s relationship to CR	0.268**	0.072**	0.340**
CR’s relationship to CG	0.227*	0.039	0.266**

The statistical results in this table are standardized. CG denotes variables pertaining to caregivers. CR denotes variables pertaining to care recipients. *p < 0.05; **p < 0.01; ***p < 0.001.

It is shown in Figure 1 and Table 3 that responsibility, caring out of love, CG’s relationship to CR, and CR’s relationship to CG not only directly affect care willingness, but also indirectly affect care willingness through the mediation of caregiver burden (except CR’s relationship to CG). By comparing the total effect of the four variables, the impact of caring out of love is the highest (0.636), the second is CG’s relationship to CR, the third is CR’s relationship to CG, and the impact of responsibility is the lowest. In other words, caring out of love and the quality of the caregiver-care recipient relationship are more helpful in predicting changes in caregiver burden, which in turn helps predict variations in care willingness, than the caregiver’s responsibility. Hypothesis 5 was testified.

## 4 Discussion

We found that it was the caregiver burden rather than the functional disability of older adults that harms family caregivers’ willingness to care. This suggests that although the functional disability of older adults is a source of caregiver burden, it is not the whole story. Therefore, a comprehensive evaluation of caregiver burden should be carried out through ZBI.

The current study found that responsibility and caring out of love were positively related to care willingness. Regardless of time and space change, family ethics will have a lasting influence on family members’ willingness to care (14–16). Many family caregivers have devoted a great deal of time and energy to looking after their loved ones (29). The maintenance of family care for disabled older adults is closely related to the responsibility and love of family caregivers for relatives, which suggest that genetic altruism still has explanatory power.

It was shown that the quality of the caregiver-care recipient relationship was positively associated with care willingness, indicating that the better the quality of the relationship, the higher the willingness to care. That is, relationship quality as a crucial contributing factor to care willingness (6, 22, 23). This phenomenon can be explained by reciprocal altruism. In the full model, the effect of relationship quality remained significant (only CR’s relationship to CG), while the effect of responsibility and love was no longer significant. This further indicates that reciprocal altruism has more explanatory power for family caregiver’s willingness to care in transition China.

Another essential finding of this study was that, path analysis not only fully demonstrated the impact of caregiver burden, responsibility, caring out of love, and the quality of the caregiver-care recipient relationship on care willingness, but also found that these independent variables were at different levels, that is, caregiver burden also acted as a mediator. Responsibility, caring out of love, and relationship quality directly and through the mediation of caregiver burden indirectly affected care willingness. This is probably because family caregivers’ burden will be reduced, and their care willingness be enhanced when their basic psychological needs are met. Ryan and Deci (30) argued that human beings have three basic psychological needs, namely, autonomy, competence, and relatedness. Among them, relatedness is a kind of belonging need, which refers to people’s universal tendency to communicate with, relate to and care for others. Since responsibility, caring out of love, and a good relationship between family caregivers and older people can satisfy family caregivers’ basic psychological needs (mainly relatedness) to a certain extent, some family caregivers’ willingness to care is higher.

With the deepening of China’s population aging, it is necessary to build a perfect old-age security system to support older people. In addition to institutional care and community care, family care is still playing a vital role. Caregiver burden and care willingness are the determinants of the sustainable development of family care, and the latter is more important than the former to some extent. If family caregivers are unwilling to care for older persons, even if they can, some older people will be in a tragic situation. Thus, policies should promote family caregivers’ willingness to care in China.

Firstly, institutional care and community care should be developed to reduce caregiver burden. Based on regular caregiver burden assessments, interventions should be given to those with a heavy burden to prevent the deterioration of caregivers’ physical and mental health, career development, financial status, and social interaction, as well as a series of other negative consequences. At the same time, we should implement a comprehensive and flexible caregiver support strategy, including respite services, training/counseling, support groups, improved relationships, flexible work arrangements, financial compensation, support for exceptional caregivers, etc.

Secondly, in transitional China, responsibility and love for old parents can still maintain family care, but the negative impact of the heavy caregiver burden on family caregivers is amplified by the individualization of society. In this case, it is more necessary to build a good family relationship, including the spouse relationship and parent–child relationship. For example, through the intervention of social workers, we can know the quality of the caregiver-care recipient relationship, and provide targeted professional services (mainly family social work). Family meetings can also be held to promote a rational division of caregiving within the family to alleviate conflicts among family members arising from caring for older people.

Thirdly, family ethics should be advocated. Family ethics is a valuable cultural resource in China that helps strengthen family members’ sense of responsibility towards older relatives. In the context of social transition, people may have different understandings of family ethics. Thus, traditional family ethics should be inherited and developed in new forms to increase family caregivers’ willingness to care for their older relatives.

Based on a sampling survey in Shanghai, this study has made some innovations. First, so far, there has been little discussion on family caregivers’ willingness to care and its contributing factors, and even less studies have been conducted in China. Using the unique sample from China, this study examined what factors influenced care willingness in contemporary China. Second, based on the theory of altruism, this study put forward some hypotheses and found that reciprocal altruism presented by the quality of the caregiver-care recipient relationship has a significantly positive impact on family caregivers’ willingness to care. Finally, the present study found that caregiver burden acted as a mediator between responsibility, caring out of love, the quality of the caregiver-care recipient relationship, and care willingness, which is helpful for us to understand the complex mechanisms that influence family caregivers’ willingness to care.

Although this unique Chinese sample contributes to examining a crucial issue in the field of family care in contemporary China, considering the number and composition of China’s older adults, the sample size was relatively small, only from Shanghai, and therefore underrepresented. The survey findings can only be extrapolated to Shanghai, not the whole country. Imperfect measurement was the second shortcoming of this study. For instance, it was not accurate to measure the quality of the caregiver-care recipient relationship with just one question. Thirdly, given the cross-sectional design, the findings can only explain the correlation between influencing factors and outcome variables (willingness to care) rather than causality. To address these limitations, first, a national survey should be carried out so that the sample can reflect the heterogeneity of respondents and the findings can be extrapolated to the population as a whole. Second, the measurement of critical independent variables should be improved to explain care willingness accurately. Third, longitudinal research is also necessary to explore the causal relationship.

## 5 Conclusion

In East Asian societies, family members are expected to take care of older relatives. However, as the family size is reducing and the burden on family caregivers is increasing, meanwhile, to fully enjoy their lives, some family members have given up or intend to give up their care for older adults. But, Chinese society still assumes that family members can and will care for their older relatives without much support, which may trigger a potential nursing crisis. Exploring family caregiver’s willingness to care from the perspective of altruism can make us effectively predict future family care changes and timely take measures to support family care.

## Data availability statement

The data analyzed in this study is subject to the following licenses/restrictions: the data that support the findings of this study are available from the corresponding author upon reasonable request. Requests to access these datasets should be directed to S-YH, hanshaoyong@zuaa.zju.edu.cn.

## Ethics statement

The studies involving human participants were reviewed and approved by the East China University of Political Science and Law Institutional Review Board (Protocol #16BSH137).

## Author contributions

JL was responsible for conception and article writing. S-YH was responsible for data mining. J-CZ and C-HX were responsible for scientific supervision. All authors contributed to the article and approved the submitted version.
